# Diagnostic Performance of an Antigen Test with RT-PCR for the Detection of SARS-CoV-2 in a Hospital Setting — Los Angeles County, California, June–August 2020

**DOI:** 10.15585/mmwr.mm7019a3

**Published:** 2021-05-14

**Authors:** Auguste Brihn, Jamie Chang, Kelsey OYong, Sharon Balter, Dawn Terashita, Zach Rubin, Nava Yeganeh

**Affiliations:** ^1^Epidemic Intelligence Service, CDC; ^2^Los Angeles County Department of Public Health, California; ^3^CHA Hollywood Presbyterian Medical Center, California.

Prompt and accurate detection of SARS-CoV-2, the virus that causes COVID-19, has been important during public health responses for containing the spread of COVID-19, including in hospital settings (*1*–*3*). In vitro diagnostic nucleic acid amplification tests (NAAT), such as real-time reverse transcription–polymerase chain reaction (RT-PCR) can be expensive, have relatively long turnaround times, and require experienced laboratory personnel.* Antigen detection tests can be rapidly and more easily performed and are less expensive. The performance^†^ of antigen detection tests, compared with that of NAATs, is an area of interest for the rapid diagnosis of SARS-CoV-2 infection. The Quidel Sofia 2 SARS Antigen Fluorescent Immunoassay (FIA) (Quidel Corporation) received Food and Drug Administration Emergency Use Authorization for use in symptomatic patients within 5 days of symptom onset (*4*). The reported test positive percentage agreement^§^ between this test and an RT-PCR test result is 96.7% (95% confidence interval [CI] = 83.3%–99.4%), and the negative percentage agreement is 100.0% (95% CI = 97.9%–100.0%) in symptomatic patients.^¶^ However, performance in asymptomatic persons in a university setting has shown lower sensitivity (*5*); assessment of performance in a clinical setting is ongoing. Data collected during June 30–August 31, 2020, were analyzed to compare antigen test performance with that of RT-PCR in a hospital setting. Among 1,732 paired samples from asymptomatic patients, the antigen test sensitivity was 60.5%, and specificity was 99.5% when compared with RT-PCR. Among 307 symptomatic persons, sensitivity and specificity were 72.1% and 98.7%, respectively. Health care providers must remain aware of the lower sensitivity of this test among asymptomatic and symptomatic persons and consider confirmatory NAAT testing in high-prevalence settings because a false-negative result might lead to failures in infection control and prevention practices and cause delays in diagnosis, isolation, and treatment.

During a period of high community COVID-19 prevalence,** the Los Angeles County Department of Public Health collaborated with hospital A, a tertiary medical center serving a large urban population in central Los Angeles, to evaluate the performance of the Quidel Sofia 2 SARS Antigen FIA (antigen test) compared with that of the Fulgent COVID-19 RT-PCR (Fulgent Genetics) (RT-PCR test) for screening of all patients admitted to the hospital through the ED during June 30–August 31. Admitting orders included requests for both tests to enable prompt inpatient cohorting. Each admitted patient had two simultaneously collected samples for SARS-CoV2 testing by ED nursing staff members: an anterior nasal swab successively swabbing both nostrils with one swab and a nasopharyngeal swab. Nasopharyngeal swab specimens were processed and sent by courier to a Clinical Laboratory Improvement Amendments–certified laboratory for RT-PCR testing. Results were available 24–48 hours after specimen collection. Test cycle threshold (Ct) values for N1 and N2 nucleocapsid viral gene targets were reported. N1 and N2 targets with Ct values <40 were used to define a positive RT-PCR result, per manufacturer instructions.^††^ Because differences between N1 and N2 targets were negligible, for this analysis, N1 target Ct values were used. The anterior nasal swab specimens were processed for antigen testing using calibrated Sofia 2 analyzers in the ED.

The RT-PCR test was used as the standard. Results were considered concordant if they were positive for both tests or negative for both, and discordant if one was positive and the other was negative. Persons were categorized as having COVID-19–compatible symptoms if they had a temperature ≥100.4°F (38°C) at triage, or reported respiratory distress, shortness of breath, cough, flu-like symptoms, nausea, vomiting, diarrhea, or headache. Signs and symptoms (ED chief complaints and vital signs) were categorized into those more commonly reported by COVID-19 patients (*6*) (i.e., fever, respiratory distress or shortness of breath, and cough) and those less commonly reported (i.e., flu-like symptoms, nausea or vomiting, diarrhea, and headache). Symptoms were retrospectively ascertained through medical record abstraction using the ED triage assessment. Hospital service codes and vital signs were evaluated for patients without an ED chief complaint. Patients who went to a non-ED location (e.g., labor and delivery), might not have an ED chief complaint and were classified as asymptomatic for this analysis. Additional information regarding symptoms was obtained from the hospital’s electronic medical records system for patients with discordant antigen and RT-PCR test results.

Data were managed and analyzed using SAS software (version 9.4; SAS Institute). Sensitivity, specificity, negative predictive value, and positive predictive value were calculated for antigen testing and compared with those of RT-PCR. N1 Ct values for antigen-positive and antigen-negative symptomatic and asymptomatic groups were compared using t-tests; p-values <0.05 were considered statistically significant. Signs and symptoms, demographic characteristics, and underlying medical conditions for the group of patients with discordant results were compared using chi-square or Fisher’s exact tests. Odds ratios were calculated for each of the more common or less common symptoms and overall. This investigation was reviewed by the Los Angeles County Institutional Review Board and CDC and was conducted consistent with applicable federal law and CDC policy.^§§^

During June 30–August 31, hospital A tested 2,039 patients admitted through the ED with paired antigen and RT-PCR tests. Median patient age was 56 years (range = 16–107 years); 1,126 (55%) were female, and 913 (45%) were male. The mean test turnaround time for RT-PCR was 28.2 hours. Overall, 307 (15%) patients had COVID-19–compatible symptoms (Table 1). Among the 307 symptomatic patients, 120 (39%) had a positive test result by either test, including 52 (17%) by antigen and 68 (22%) by RT-PCR. Positive test result by both the antigen and the RT-PCR tests were reported for 49 (16%) patients. Mean N1 Ct values were significantly lower among patients with a positive antigen result (mean Ct = 21.3) than among patients with a negative antigen result (mean Ct = 28.5; p<0.001).

**TABLE 1:** Characteristics* of the Quidel Sofia 2 SARS Antigen Fluorescent Immunoassay test among symptomatic and asymptomatic persons admitted to a tertiary medical center through the emergency department (N = 2,039) — Los Angeles County, California, June 30–August 31, 2020

Test diagnostic characteristic	All patients (N = 2,039)	Symptomatic patients (n = 307)	Asymptomatic patients (n = 1,732)	p-value^†^
Positive RT-PCR test results, no. (%)	149 (7.3)	68 (22.2)	81 (4.7)	—
Positive antigen test results, no. (%)^§^	110 (5.4)	52 (16.9)	58 (3.4)	—
Sensitivity of antigen test, % (95% CI)	65.8 (57.6–73.3)	72.1 (61.4–82.7)	60.5 (49.9–71.1)	0.16
Specificity of antigen test, % (95% CI)	99.4 (98.9–99.7)	98.7 (97.3–100.0)	99.5 (99.1–99.8)	0.19
Positive predictive value of antigen test, % (95% CI)	89.1 (81.7–94.2)	94.2 (87.9–100.0)	83.0 (75.2–93.8)	0.13
Negative predictive value of antigen test, % (95% CI)	97.4 (96.5–98.0)	92.6 (89.3–95.8)	98.1 (97.4–98.7)	<0.001

**Abbreviations:** CI = confidence interval; RT-PCR = reverse transcription–polymerase chain reaction.

* Quidel Sofia 2 SARS Antigen Fluorescent Immunoassay test characteristics (sensitivity, specificity, positive predictive value, and negative predictive value) were based on comparison with the Fulgent COVID-19 RT-PCR test

^†^ Chi-square and Fisher’s exact p-value comparing symptomatic patients with asymptomatic patients.

^§^ At hospital A, the Quidel Sofia 2 SARS Antigen Fluorescent Immunoassay was used for qualitative detection of nucleocapsid protein from SARS-CoV-2.

Among the 1,732 asymptomatic patients, 139 (8%) had a positive test result by either test (58 [3%] by antigen and 81 [5%] by RT-PCR). Mean N1 Ct values did not differ significantly between samples from patients who were symptomatic (mean Ct = 23.5) and those who were asymptomatic (mean Ct = 23.9). Among asymptomatic and symptomatic patients, the specificity of the antigen test was 99.5% and 98.7%, respectively, and the sensitivity was 60.5% and 72.1%, respectively. The diagnostic performance between the two groups did not differ significantly, with the exception of negative predictive value (p<0.001). Sensitivity of the discordant antigen test results from patients who were symptomatic and asymptomatic was assessed across a range of Ct values. Antigen test sensitivity increased in symptomatic and asymptomatic persons as N1 Ct values decreased (sensitivity 75% for Ct ≤30 and sensitivity 90.7% for Ct ≤25).

RT-PCR–positive and antigen-positive test results were compared with patients’ signs and symptoms at the time of admission. Symptoms associated with a positive RT-PCR test result included fever, respiratory distress or shortness of breath, cough, and flu-like symptoms (Table 2). Shortness of breath was the most commonly reported symptom among persons with a positive RT-PCR test result (28%) and among both discordant groups (RT-PCR–positive/antigen-negative = 39%; RT-PCR–negative/antigen-positive = five of 12 patients) (Table 3). No COVID-19–compatible symptoms occurred in 27 (53%) patients with RT-PCR positive/antigen-negative test results and six of 12 patients with RT-PCR negative/antigen-positive test results. Some patients with RT-PCR–positive/antigen-negative test results had underlying medical conditions recorded in medical records (10% reporting having diabetes and 18% having hypertension) and were at higher risk for severe COVID-19–associated illness.^¶¶^

**TABLE 2:** Frequency and odds ratios for RT-PCR–positive results among patients admitted to hospital through a tertiary medical center emergency department, by chief complaint (N = 1,667)* — Los Angeles County, California, June 30–August 31, 2020

Patient’s chief complaint	No. (%)		OR (95% CI) for RT-PCR–positive results^†^
	RT-PCR–positive results (n = 138)	RT-PCR–negative results (n = 1,529)	
**More common COVID-19–like signs and symptoms**			
Fever/Chills	11 (8.0)	31 (2.0)	4.2 (2.1–8.5)
Respiratory distress/Shortness of breath	39 (28.0)	150 (10.0)	4.1 (2.8–6.1)
Cough	6 (4.0)	8 (0.5)	9.9 (3.4–28.8)
**Less common signs and symptoms**			
Flu-like symptoms	10 (7.0)	5 (0.3)	27.1 (9.1–80.6)
Nausea/Vomiting	1 (0.7)	29 (2.0)	0.4 (0.1–3.2)
Diarrhea	1 (0.7)	5 (0.3)	2.5 (0.3–21.9)
Headache	0 (—)	11 (0.7)	0 (—)
**Met case definition^§^**	68 (49.0)	239 (16.0)	5.2 (3.7–7.5)

**Abbreviations:** CI = confidence interval; OR = odds ratio; RT-PCR = reverse transcription–polymerase chain reaction.

* 372 patients (11 RT-PCR–positive and 361 RT-PCR–negative) with missing emergency department chief complaint data were excluded.

^†^ Among patients with and without symptoms.

**^§^** Case was defined as symptomatic if patient had a chief complaint of more common or less common COVID-19–compatible signs and symptoms.

**TABLE 3:** Characteristics of patients admitted to hospital through a tertiary medical center emergency department with discordant SARS-CoV-2 antigen and RT-PCR test results* (N = 63)^†^ — Los Angeles County, California, June 30–August 31, 2020

Discordant group characteristic	No. (%)		
	RT-PCR–positive^§^/Antigen-negative (n = 51)	RT-PCR–negative/Antigen-positive^¶^ (n = 12)	Total (N = 63)
Signs and symptoms at emergency department admission			
Fever/Chills	18 (35)	1 (8)	**19 (30)**
Cough	15 (29)	0 (0)	**15 (24)**
Shortness of breath	20 (39)	5 (42)	**25 (40)**
Fatigue	6 (12)	0 (—)	**6 (10)**
Muscle aches	9 (18)	0 (—)	**9 (14)**
Headache	0 (0)	1 (8)	**1 (2)**
Loss of taste or smell	1 (2)	1 (8)	**2 (3)**
Sore throat	3 (6)	0 (—)	**3 (5)**
Congestion	5 (9)	0 (—)	**5 (8)**
Nausea/Vomiting	7 (13)	1 (8)	**8 (13)**
Diarrhea	5 (10)	0 (—)	**5 (8)**
No symptoms**	27 (53)	6 (50)	**—**
Temperature >100.4°F (38°C)	5 (10)	5 (42)	**5 (8)**
**Demographic characteristic**			
**Sex**			
Female	25 (49)	8 (67)	**35 (56)**
Male	24 (47)	4 (33)	**28 (44)**
**Race** ^††^			
Asian	7	5	**12**
White	6	—	**6**
Black	3	1	**4**
Other	32	6	**41**
Unknown	6	—	**—**
**Age, yrs, mean (range)**	59 (20–98)	67 (28–100)	**60 (21–100)**
**Underlying medical condition**			
Diabetes	5 (10)	1 (8)	**6 (10)**
Obesity	2 (4)	0 (—)	**2 (3)**
Hypertension	9 (18)	2 (17)	**11 (18)**
Heart disease	2 (4)	3 (25)	**5 (8)**

**Abbreviation:** RT-PCR = reverse transcription–polymerase chain reaction.

* False negative = antigen-negative and RT-PCR–positive; false positive = antigen-positive and RT-PCR–negative.

^†^ 2,039 patients admitted through the emergency department were tested with paired SARS-CoV-2 antigen and RT-PCR tests.

^§^ The Fulgent COVID-19 by RT-PCR test, a real-time RT-PCR test intended for the qualitative detection of nucleic acid from SARS-CoV-2 in upper and lower respiratory specimens, was used.

^¶^ The Quidel Sofia 2 SARS Antigen Fluorescent Immunoassay was used for qualitative detection of the SARS-CoV-2 nucleocapsid protein.

** No symptoms identified through individual medical chart abstraction.

^††^ Ethnicity data were not collected for this analysis.

## Discussion

In this analysis of RT-PCR and antigen testing of asymptomatic and symptomatic patients at the time of a tertiary hospital admission through the ED, the sensitivity of the Quidel Sofia 2 SARS Antigen FIA test was 66% (72% and 61% in symptomatic and asymptomatic patients, respectively) using the Fulgent COVID-19 RT-PCR test as the standard; specificity was high overall (>99%). The antigen test’s sensitivity increased in specimens with lower Ct values, consistent with higher virus titers in the specimen. Proper interpretation of the antigen test results should consider the patient’s signs, symptoms, and exposure history, the prevalence of COVID-19 in the community, and the test’s performance characteristics.*** The lower sensitivity of antigen tests compared with RT-PCR testing supports the strategy of using a more sensitive NAAT test if there is high clinical suspicion for COVID-19. COVID-19–compatible symptoms in this study were associated with positive RT-PCR test results. A positive antigen test result with a high pretest probability, either because of symptoms, exposure to an active case, or residence in an area of high community prevalence, could enable early isolation and receipt of medical care. This analysis did not identify any statistical difference between N1 Ct values in the study samples collected from symptomatic and asymptomatic persons. Findings indicate that although sensitivity of the antigen test does increase with lower Ct values, sensitivity is still lower at Ct values <30 and even at Ct values <25 in symptomatic and asymptomatic persons.

The findings in this report are subject to at least four limitations. First, this community and tertiary medical center represent a convenience sample and are not representative of all U.S. community and medical center settings. Second, data regarding any COVID-19–compatible symptoms reported were not collected beyond the ED chief complaint for the concordant group; therefore, the number of symptomatic persons might be underestimated. Third, exposure history was not evaluated. Finally, RT-PCR is an imperfect standard for comparison because it detects the presence of viral RNA, which includes “dead” virus and might not be correlated with transmission. 

Overall, this evaluation of the performance of a rapid antigen test among symptomatic and asymptomatic persons suggests cautious interpretation of rapid antigen test results given its lower sensitivity. A false-negative antigen test result in health care settings might lead to failures in infection control and prevention practices and cause delays in diagnosis, isolation, and treatment. Persons with COVID-19–compatible symptoms and negative Quidel Sofia 2 SARS Antigen FIA antigen test results should have an additional sample confirmed with a NAAT test. While awaiting confirmation, measures to prevent SARS-CoV-2 transmission are recommended, including the use of personal protective equipment, source control for the patient, adherence to infection prevention protocols, and avoidance of cohorting these patients with others who do not have confirmed or suspected COVID-19 infection.^†††^

**Summary**

**What is already known about this topic?**

Prompt and accurate diagnosis of SARS-CoV-2 infection is critical to containing the spread of COVID-19 in a hospital setting.

**What is added by this report?**

The Quidel rapid antigen test had lower sensitivity in both asymptomatic (60.5%) and symptomatic (72.1%) patients but a high specificity (98.7% and 99.5% for symptomatic and asymptomatic patients, respectively) when compared with the reverse transcription–polymerase chain reaction (RT-PCR) test.

**What are the implications for public health practice?**

Antigen tests have lower sensitivity compared with RT-PCR; negative antigen test results in persons with symptoms should be confirmed with an RT-PCR test, because a false-negative result might lead to failures in infection control and prevention practices and cause delays in diagnosis, isolation, and treatment.

## Footnotes

*
https://www.medrxiv.org/content/10.1101/2020.06.22.20136309v3

† Test performance includes sensitivity, specificity, positive predictive values, and negative predictive value.

§ The estimate for positive percentage agreement and negative percentage agreement is used in place of sensitivity in the absence of a reference standard test for comparison.

¶
https://www.quidel.com/sites/default/files/product/documents/EF1438905EN00.pdf

**
http://dashboard.publichealth.lacounty.gov/covid19_surveillance_dashboard/

†† https://www.fda.gov/media/138150/download

§§ 45 C.F.R. part 46.102(l)(2), 21 C.F.R. part 56; 42 U.S.C. Sect. 241(d); 5 U.S.C. Sect. 552a; 44 U.S.C. Sect. 3501 et seq.

¶¶
https://www.cdc.gov/coronavirus/2019-ncov/need-extra-precautions/people-with-medical-conditions.html

***
https://www.cdc.gov/coronavirus/2019-ncov/lab/resources/antigen-tests-guidelines.html

†††
https://www.cdc.gov/coronavirus/2019-ncov/hcp/using-ppe.html
